# Acute coronary syndromes across the lifespan of women

**DOI:** 10.1038/s44325-026-00126-5

**Published:** 2026-05-25

**Authors:** Teodora Donisan, Grace Hagan, Marysia S. Tweet, Malcolm R. Bell, Sharonne N. Hayes

**Affiliations:** 1 Department of Cardiovascular Medicine, Mayo Clinic, Rochester, MN USA; 2 Department of Internal Medicine, Mayo Clinic, Rochester, MN USA

**Keywords:** Cardiology, Diseases, Health care, Medical research, Risk factors

## Abstract

Acute coronary syndromes (ACS) in women differ from those in men, with age-specific variations in etiology, presentation, and outcomes. Women face distinct risk factors, from autoimmune conditions to pregnancy-associated complications, to microvascular dysfunction. Missed and delayed diagnosis and treatment, and underrepresentation in clinical research compound these risks. This review outlines the evolving mechanisms of ACS in women and calls for tailored, evidence-based strategies to improve diagnosis, prevention, and care.

## Introduction

Acute coronary syndromes (ACS) remain a leading cause of morbidity and mortality among women worldwide, with over 3.2 million women in the United States living with a history of myocardial infarction (MI) and more than 41,000 annual deaths attributed to ACS^1,2^. Women compared to men with ACS face biases and knowledge gaps that result in missed and late diagnoses, undertreatment, and poorer outcomes^3^. Despite increasing awareness, significant sex- and gender-based disparities persist in our understanding of the epidemiology, diagnosis, management, and outcomes of ACS. These disparities reflect biological and pathophysiological differences in disease manifestation; evidence gaps due to the longstanding lack of focus on heart disease in women; and historical exclusion and underrepresentation of women from cardiovascular clinical trials, especially those of reproductive age and the elderly.

Sex differences in ACS encompass a spectrum of mechanisms, ranging from coronary atherosclerosis, spontaneous coronary artery dissection (SCAD), vasospasm, and coronary microvascular dysfunction, to non-obstructive plaque rupture or erosion and embolic phenomena^2^. The non-atherosclerotic and non-obstructive mechanisms are more prevalent in women and frequently missed with traditional diagnostic algorithms^4–7^. Women’s symptoms are also more likely to include non-chest pain symptoms, especially at younger and older ages. Women more commonly exhibit non-obstructive coronary artery disease (CAD) on angiography despite ischemic presentations, contributing to diagnostic uncertainty and therapeutic inertia^7^.

Importantly, ACS risks and phenotypes are not static throughout a woman’s life. Unique exposures such as pregnancy and its complications, hormonal transitions, autoimmune disease, and breast cancer therapies modulate cardiovascular risk at different life stages. Traditional risk factors also exert differential effects by sex. Therefore, a lifespan-based approach is critical to appreciating the nuanced and evolving interplay of risk, biology, and presentation that contributes to ACS in women.

Herein, unique diagnostic considerations in the care of women with ACS across the lifespan will be discussed. This will incorporate consideration of gender biases and racial and ethnic disparities in disease burden and recognition, cardiovascular risk factors, and a synthesis of current knowledge on ACS in women, from pediatric and adolescent presentations to reproductive-age, menopausal, and older adult stages. We highlight age-specific risk factors, etiologies, diagnostic considerations, and treatment challenges, with the goal of informing tailored, equitable, and evidence-based care for women across all ages.

## Mechanisms and modifiers unique to women

### Unique considerations across the lifespan

#### Gender biases in ACS recognition and management

Although women with ACS present with chest pain nearly as frequently as men, and chest pain remains the predominant symptom of women presenting with ACS, they are more likely to experience a delay to diagnosis^8^. Women comprise the majority of emergency visits for chest pain and may experience a higher number of presenting symptoms than men for comparative degrees of CAD^9^. Women may be less likely to seek emergent care due to a lack of awareness of myocardial infarction (MI) symptoms, caregiving responsibilities, or other barriers, whereas clinicians and emergency responders may discount or attribute ACS symptoms in women to noncardiac etiologies^10^.

Biases may be even more prominent in younger women, in whom there is known disproportionate prevalence of non-atherosclerotic causes of ACS, such as SCAD, and lower prevalence of traditional cardiovascular risk factors, compared to their male counterparts^2,7^. In the VIRGO (Variation In Recovery: Role of Gender on Outcomes of Young AMI Patients) and YOUNG-MI registries, women under age 55 with MI were less likely to undergo coronary angiography or revascularization and had higher post-MI mortality^11,12^. Women with STEMI received fewer reperfusion therapies and experienced longer delays to percutaneous coronary intervention (PCI) or fibrinolysis compared to men^13^.

Recognizing these gaps, the joint committee of the American College of Cardiology (ACC) and American Heart Association (AHA) Chest Pain Guidelines include class I indications to (1) always consider cardiac causes as differential for women presenting with chest pain and (2) emphasize accompanying symptoms when obtaining a history from women presenting with chest pain^14^. An early invasive strategy in high-risk women should be employed, with appropriate additional invasive and non-invasive testing if coronaries are normal or have minimal CAD^2^.

#### Ethnic and racial differences and disparities in cardiovascular care

CVD is the leading cause of death across all racial and ethnic groups among women in the United States, but disparities persist in disease burden, recognition, and treatment. These ethnic and racial disparities are rooted in structural inequities, including differential access to care, food insecurity, housing instability, and chronic exposure to racism and stress^1^. Non-Hispanic Black women have the highest burden of cardiovascular disease prevalence among US women (59%), including the highest prevalence of hypertension (58.4%), obesity (57.9%), and diabetes (13.3%), and also experience worse^1^. Similarly, the prevalence of CVD in US Hispanic women approaches 40% and is the leading cause of mortality in this patient population^1^. Hispanic women are more likely to have comorbid conditions such as hyperlipidemia, hypertension, and obesity when compared to non-Hispanic white women, and much of this can be attributed to social determinants of health and generational psychological burden^15^. American Indian and Alaska Native women, while absent or significantly underrepresented in clinical studies, carry a disproportionately high burden of CVD morbidity and mortality, driven primarily by diabetes, with a threefold higher prevalence than in White women^16^, compounded by high rates of hypertension, obesity, and poverty^1^ that occur earlier in life. The intersection of these racial and ethnic disparities with the impact of sex and gender factors reflects social determinants of health and cumulative life course exposures. Efforts to reduce inequities must incorporate community-specific strategies, improve data collection, and prioritize culturally competent care^17^.

## Risk factors for acute coronary syndrome among women—unique, disproportionate, and different

### Risk factors unique to women

**Fig. 1:**
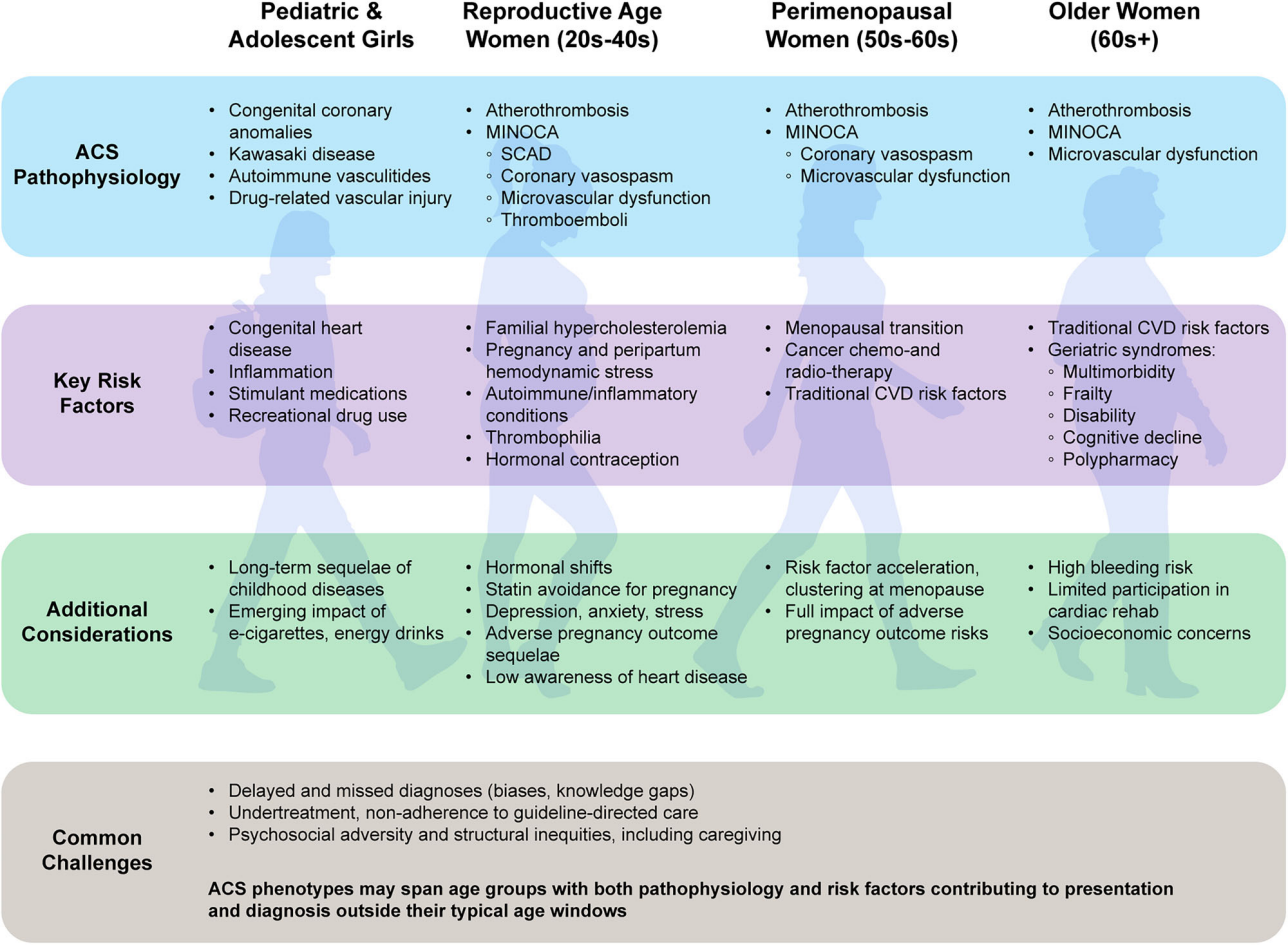
Acute coronary syndromes in women across the lifespan. Representative ACS phenotypes, key risk factors, and clinical modifiers by age group in women, from childhood through older adulthood. ACS acute coronary syndrome, CVD cardiovascular disease, FH familial hypercholesterolemia, MINOCA myocardial infarction with non-obstructive coronary arteries, SCAD spontaneous coronary artery dissection, SLE systemic lupus erythematosus.

Women experience both traditional and non-traditional cardiovascular risk factors, but their expression, impact, and clinical consequences often differ from those in men (Tables 1 and 2). Understanding these variations is essential to delivering age-, gender-, and sex-specific care (Fig 1).

**Table 1:** Therapeutic domains for the management of cardiovascular disease in women

Therapeutic domain	Key considerations in women	Age or subgroup specifics
Statins	Under-prescription, pregnancy-related interruptions	Reproductive-age women
DAPT	Shorter duration may be preferred	SCAD, older women
Anticoagulation	Caution during pregnancy, atrial fibrillation	Peripartum, older women
Menopausal Hormone Therapy	Risk outweighs any cardioprotective benefit, increased thrombotic risk	Late postmenopausal women
Combined hormonal contraception	Estrogen-containing products increase thrombotic risk in predisposed patients	SLE, thrombophilia, and active smokers
Cardiac Rehabilitation	Under-referred, lack of individualized programs, logistical and psychosocial barriers	All age groups
Pregnancy after ACS	Requires pre-conception risk stratification and cardio-obstetrics collaboration	Post-SCAD, prior thrombotic ACS, established CAD

This table describes medication considerations for women with associated age or subgroup specifics.

*ACS* acute coronary syndrome, *CAD* coronary artery disease, *DAPT* dual antiplatelet treatment, *SLE* systemic lupus erythematosus, *SCAD* spontaneous coronary artery dissection.

**Table 2:** Unique cardiovascular considerations regarding ACS in women across the lifespan

Age group	Traditional risk factors	Unique considerations in women
Pediatric + Adolescent (10–19 years)	• Congenital heart disease • Cigarette/Tobacco use • Stimulants (dextroamphetamine) • Childhood obesity	• Autoinflammatory conditions (Kawasaki’s, SLE) • Early menarche • Body image concerns/dieting behaviors • Primary amenorrhea • PCOS • Combined hormonal contraceptives
Early Adulthood (20–39 years)	• Primary hypertension • E-cigarette/Tobacco use • Prescribed + illicit stimulants • Obesity	• Pregnancy-related cardiovascular risk (gestational diabetes, gestational hypertension, preeclampsia, HELLP syndrome, preterm delivery, peripartum cardiomyopathy, pregnancy loss) • Infertility + hormonal delivery for ovulation induction • Autoimmune conditions (SLE, APLS)
Middle Age, Peri-/Postmenopausal (40–64 years)	• Primary hypertension • E-cigarette/Tobacco use • Diabetes Mellitus • Obesity • Hyperlipidemia	• Premature ovarian failure • Breast cancer therapy (trastuzumab, anthracyclines) • Perimenopause transition • Hormone replacement therapy

*APLS* antiphospholipid syndrome, *PCOS* polycystic ovarian syndrome, *SLE* systemic lupus erythematosus.

Cardiovascular risk factors unique to women begin prior to pregnancy and menopause. Early reproductive milestones such as menarche and menstrual cycle regularity offer insights into cardiometabolic health. Early and late menarche, menstrual cycle irregularity, and polycystic ovary syndrome are associated with adverse profiles including insulin resistance, dyslipidemia, and elevated blood pressure, and have been linked to increased long-term cardiovascular risk^18^. These should be considered in cardiovascular risk assessment starting in adolescence.

#### Pregnancy and pregnancy complications

The experience of pregnancy itself, a foundational sex difference, causes numerous hormonal and physiologic changes that are relevant to the cardiovascular system, including changes in circulating blood volume, systemic vascular resistance, neovascularization, blood pressure, and thrombotic risk, among others. The physiological stress of pregnancy can reveal underlying vascular or metabolic dysfunction, and adverse pregnancy outcomes often precede the development of overt CVD^19–21^. Adverse pregnancy outcomes, including hypertensive disorders of pregnancy (preeclampsia, eclampsia, gestational hypertension), gestational diabetes, intrauterine growth restriction, placental abruption, and preterm birth, are independently associated with a 2- to 3-fold increased risk of future CVD and ACS^22,23^. Additionally, women who have myocardial infarction and a history of preeclampsia are more likely to die, develop cardiogenic shock, and left ventricular dysfunction compared to those without preeclampsia. These risks persist for decades after delivery and are only partially explained by the development of traditional risk factors such as hypertension and diabetes^24^. Pregnancy-associated ACS is a rare but severe entity, most commonly occurring in the third trimester or early postpartum period, and frequently due to SCAD, atherothrombosis, or de novo thrombosis, in the absence of atherosclerosis^25^.

#### Menopause and the perimenopausal transition

Menopause is a critical inflection point. The loss of estrogen leads to impaired nitric oxide-mediated vasodilation, increased sympathetic tone, and adverse changes in lipids, blood pressure, and body composition^26,27^. Risk factor clustering occurs rapidly during the menopausal transition and may partially explain the increased incidence of atherosclerotic ACS in the postmenopausal period. Menopause-associated microvascular dysfunction is also a contributor to angina with non-obstructive coronary arteries (ANOCA) and ischemia with non-obstructive coronary arteries (INOCA)^28,29^.

The cardiovascular implications of menopause vary by its type and timing. Natural menopause is associated with a gradual estrogen level decline and vascular changes. Early menopause (before age 45) and premature ovarian insufficiency (before age 40) confer a significantly higher risk of CVD, particularly CAD and all-cause mortality, presumably due to the loss of favorable effects of estrogen on arteries and metabolism^30,31^. This increased risk is even more pronounced in women who undergo surgical menopause, even with ovarian conservation^32^, especially without subsequent estrogen therapy^31^. These distinctions underscore the importance of considering menopausal timing and etiology when assessing cardiovascular risk.

### Disproportionate burden of risk factors

Beyond sex- and gender-specific risk, several medical diagnoses that predominantly affect women also confer a higher CVD risk.

#### Autoimmune and inflammatory conditions

Women are disproportionately affected by systemic autoimmune conditions such as systemic lupus erythematosus (SLE), rheumatoid arthritis (RA), systemic sclerosis, antiphospholipid syndrome (APLS), and systemic vasculitides, which are potent, non-traditional risk factors for ACS in women. These conditions confer 2–5× increased risk of premature CAD and promote atherogenesis through chronic inflammation, immune-mediated endothelial injury, and accelerated atherosclerosis, oxidative stress, impaired vascular repair, and prothrombotic states^33^. Notably, younger women with SLE have a 50-fold higher risk of MI than age-matched controls^34^. Even with disease-modifying therapies, patients remain at higher cardiovascular risk compared to the general population^35^. Current guidelines emphasize the need for heightened risk awareness, but there remains a lack of specific recommendations for primary prevention in this group.

#### Cancer and cancer therapy-related cardiotoxicity and CVD risk

There is increasing evidence of a bidirectional risk relationship between CVD and cancer^36^. Cancer treatments are associated with increased long-term risks of both obstructive and non-obstructive ACS, leading to ischemic heart disease, heart failure, and arrhythmias^37^. Cardiac symptoms in this population may be misattributed to treatment-related fatigue or deconditioning, leading to delayed cardiovascular evaluation and underdiagnosis. Conversely, individuals with CVD may have an increased risk of developing cancer, possibly mediated by chronic inflammation, neurohormonal activation, or shared molecular pathways. Given the sex distribution of many cancers, particularly breast cancer, the net population-level impact of cancer therapy-related cardiovascular risk is greater in women and disproportionate in younger women compared to men^38^, underscoring the importance of sex-specific surveillance and prevention strategies.

Pertinent for women with breast cancer, CVD is a leading non-cancer cause of mortality in survivors^39^. Anthracyclines and trastuzumab can induce endothelial dysfunction, microvascular rarefaction, and myocardial injury. Breast cancer radiotherapy, particularly to the left breast, has been strongly linked to accelerated coronary atherosclerosis and increased risk of ACS that may emerge years after treatment, independent of traditional risk factors. Fortunately, modern radiation therapy techniques have markedly lessened the risk^40^.

Beyond breast cancer, malignancies such as lymphoma, head and neck cancers, and gynecologic cancers are relevant for CVD risk, particularly in mid-to-late life, and their treatments confer cardiovascular risk^41^. Thoracic radiation is strongly associated with premature CAD and ACS, due to progressive large- and small-vessel vasculopathy. The risk increases with cumulative dose and time, independent of traditional risk factors^40^. Other agents that heighten ACS risk include 5-fluorouracil, capecitabine, and taxanes, which can induce coronary vasospasm. Cisplatin has been linked to thrombosis and accelerated atherosclerosis, and vascular endothelial growth factor inhibitors and BCR-ABL tyrosine kinase inhibitors contribute to ischemic risk via hypertension and endothelial dysfunction^41^. Clinicians should recognize these risks and monitor for cardiovascular complications in women cancer survivors^42^.

#### Psychosocial adversity

Psychosocial stress and depression are more prevalent in women and independently associated with ACS incidence and outcomes, particularly in younger women^9,43,44^. Chronic caregiving stress, lower socioeconomic status, and limited access to care further exacerbate these risks^45^. Chronic stress is thought to mediate cardiovascular disease through autonomic imbalance, endothelial dysfunction, and pro-inflammatory activation^44^. Women are more likely to serve as caregivers, and high-intensity caregiving is associated with elevated cardiometabolic risk and vascular dysfunction^46^.

Young women have twice the risk of developing mental-stress-related myocardial ischemia after an MI when compared to men, the mechanism thought to be microvascular dysfunction and peripheral vasoconstriction^47^. Depression increases the risk of cardiac death by over 50% and is a strong predictor of early-onset MI^48^. Depression has been linked to autonomic dysfunction, heightened platelet reactivity, inflammation, and contributes to poorer outcomes after ACS through lower medication adherence and rehabilitation uptake^44,49,50^. Psychosocial stress and depression are a key modifiable risk factor for women of all ages, especially for those in underrepresented groups (Table 3). A recent European Society of Cardiology consensus statement on mental health in CVD provides a comprehensive review of this bidirectional relationship, including ACS and highlights the importance of considering both sex and gender in diagnosis and management of the psycho-cardio interaction^51^.

**Table 3:** Socioeconomic and psychological stressors contributing to cardiovascular risk in women

Drivers/Factors	Impact on CVD	Examples/Triggers
**Social and structural factors**		
Caregiver burden	Chronic stress, sympathetic overactivity, ischemic risk	Caring for aging patients, a chronically ill partner, and disabled children
Financial insecurity	Delayed presentation, medication non-adherence, lack of access to care, food insecurity, lack of safe exercise	Job loss, gender wage disparities, and inability to work due to caregiver responsibility
Intimate partner violence	PTSD, chronic stress, autonomic dysfunction	Recurrent violence/trauma, unsafe home, lack of financial independence to leave the relationship
Discrimination	Chronic exposure to discrimination + allostatic load, higher risk for comorbid conditions	Workspace discrimination, healthcare dismissal
**Psychological factors**		
Depression	Independent predictor of MI and poor prognosis post-MI	Predisposition to depression, peripartum depression, familial reliance, and perimenopausal changes
Anxiety disorders	High sympathetic states, tachycardia ± arrhythmias, Takotsubo cardiomyopathy predisposition	Predisposition to anxiety, acute panic attacks, and social anxiety
Social isolation/loneliness	Increases all-cause and cardiac mortality	Single-parenting, predominant male work environments

*CVD* cardiovascular disease, *MI* myocardial infarction, *PTSD* post-traumatic stress disorder.

### Traditional risk factors with a greater impact on women

Traditional cardiovascular risk factors have a more deleterious impact in women than in men, contributing to sex-based disparities in ACS incidence and outcomes^52^. Diabetes mellitus is less prevalent in women than men, but confers a 40% higher attributable risk of CVD in women when compared to men^53^. Furthermore, diabetes is present in a higher proportion of MI cases in women than men (26.8 vs 9.9%, respectively)^54^. Hypertension prevalence sharply increases in women after age 60–65; only 29% of elderly women have adequate blood pressure control compared to 41% of similarly aged men^55^. Stage 1 and 2 hypertension are stronger risk factors for ACS in women than men^56,57^. While smoking rates in women have plateaued, women who smoke face disproportionately greater cardiovascular harm compared to men. Women who smoke have a 25 percent higher relative risk of cardiovascular disease than men smokers, with a particularly elevated risk of ST-segment elevation myocardial infarction (STEMI) in women under age 50 (incidence rate ratio 13.2 vs 8.6)^58^. Smoking also eliminates the typical sex-based delay in myocardial infarction, with first MI occurring 13 years earlier in women who smoke (60.3 years) compared to non-smokers (73.4 years)^58^.

Obesity is highly prevalent in women and is increasing (40.3%), with 9.4% of women being severely obese (BMI > 40 kg/m^2^)^59^. Among non-Hispanic Black women, the prevalence (55.3%) of obesity is still the highest of any demographic group^18^. Obese women have a lower prevalence of coronary disease than obese men (28.6 vs 46.3% in those aged ≥65), which could be due to delayed onset of hypertension and dyslipidemia by 6–8 years^60^. These findings underscore the importance of earlier screening and intervention for cardiometabolic risk in women with obesity.

Women have lower rates of physical activity than men (insufficient physical activity occurred in 33.8% of women versus 28.7% of men), with this gap persisting across all age groups and increasing with age and in certain racial/ethnic and socioeconomic subgroups^61^. Low physical activity is associated with a higher risk of MI, with a stronger protective effect of exercise observed in women than in men^52^.

### Transgender women

Cardiovascular risk in sexual and gender minorities (SGM) is generally elevated due to both traditional and non-traditional CVD risk factors, although data are lacking due to low rates of inclusion of sexual orientation and gender identity (SOGI) information in clinical settings and research. Minority stress (e.g., discrimination, victimization, internalized stigma), depression, and healthcare discrimination are highly prevalent among SGM and are associated with increased rates of tobacco use, physical inactivity, obesity, and poor mental health^62,63^.

Transgender women (assigned male at birth) undergoing gender-affirming hormone therapy (GAHT) have an increased risk of both arterial and venous thromboembolic events compared to cisgender counterparts^64^. This has been shown in large retrospective studies of estrogen GAHT^65,66^; however, results from the use of testosterone GAHT are mixed^63,67^. Notably, there is a growing trend of individuals initiating gender transition later in life, often after the development of established CAD. In these patients, additional risk from exogenous estrogen may increase cardiovascular risk, including risk of MI, stroke, and venous thromboembolism, potentially compounding pre-existing atherosclerotic burden^60,68^. The importance of collecting SOGI information on all patients and applying emerging evidence to their care cannot be overemphasized.

## Acute coronary syndrome in women by life stage

### Pediatric and adolescent girls

In pediatric and adolescent girls, cardiovascular risk is shaped primarily by congenital anomalies, inflammatory disorders, and drug-related vascular injury (Table 2). Though ACS are rare in this age group, early-life exposures and conditions may establish a foundation for later CVD. Noncoronary cardiac conditions such as hypertrophic cardiomyopathy, long QT syndrome, and inherited arrhythmia syndromes, including Brugada and Wolff-Parkinson-White, are more common than ACS as contributors to sudden cardiac death in this population.

#### Congenital coronary anomalies

Congenital coronary anomalies are rare and often asymptomatic and diagnosed incidentally, but they can be clinically significant and an important cause of sudden cardiac death among young individuals. Based on angiographic data, prevalence ranges from 0.3 to 1.3% and appears somewhat higher in females^69^. These anomalies often coexist with structural congenital heart disease (CHD). It is unclear whether the frequency of malignant, interarterial, and intramyocardial course anomalies differs significantly between females and males.

Children born with CHD who survive into adulthood are at increased risk of premature CAD, likely due to interactions between congenital alterations, cumulative endothelial dysfunction, and traditional risk factors^69,70^. Some data suggest that women born with CHD may be at higher risk for CAD than males with CHD, speculated to be due to lower rates of traditional risk factors like smoking or hyperlipidemia in men with CHD when compared to women^71^. This potential shift in risk burden requires further study, as current data are limited.

#### Inflammatory and autoimmune disorders

Kawasaki disease is the most common cause of acquired heart disease in children in developed countries, with an annual U.S. incidence of 20–25 per 100,000^72^. Kawasaki is male-predominant, but long-term sequelae in females may still contribute to early CAD.

In contrast, SLE is strongly female-predominant, and pediatric-onset SLE carries a high risk for premature CAD, even in the absence of traditional risk factors^73,74^. This excess risk is attributed to chronic inflammation, cytokine dysregulation, oxidative stress, antiphospholipid antibodies, and vascular toxicity from immunosuppressive therapies^75^. Juvenile idiopathic arthritis also affects girls disproportionately and has been associated with increased arterial stiffness and subclinical atherosclerosis^76^. Additionally, childhood cancer survivors, including girls treated for Hodgkin lymphoma with mantle radiation, face increased long-term risk of CAD, valvular disease, and cardiomyopathy^77^. Despite this, there is no validated cardiac risk assessment strategy for pediatric SLE, arthritis, or cancer survivors, and limited consensus on screening or prevention, posing challenges for early intervention.

#### Medication and illicit substance-related vascular injury

Among adolescents, vascular dysfunction from medications or substances is an emerging concern. Prescription stimulants (e.g., for attention-deficit hyperactivity disorder) are widely used in adolescent populations. While generally safe, high doses or prolonged use have been associated with elevated blood pressure and increased arterial stiffness, particularly in those with predisposing risk factors^78^. Sex-specific surveillance data are limited, and it remains unclear whether cardiovascular effects differ between girls and boys.

Beyond prescription stimulants, other substances increasingly used by adolescents may contribute to vascular dysfunction and elevated cardiovascular risk. Epidemiological data demonstrate that recreational substance use (e.g., tobacco, alcohol, cocaine, amphetamines, cannabis) is independently associated with premature and extremely premature atherosclerotic CVD, and the magnitude of risk is greater among women than men^79^. Cannabis smoking, vaping, or ingestion, while often perceived as benign, has been associated with coronary vasospasm, arrhythmias, and increased myocardial oxygen demand, particularly when combined with tobacco or stimulants^80^. These effects are not sex-specific, but the epidemiological evidence indicates that the adverse cardiovascular impact may be more pronounced in women^81^.

Cocaine is clearly linked to ACS risk via coronary vasospasm, endothelial injury, and prothrombotic effects^82^. Women with cocaine use disorder have a significantly higher risk of cardiovascular hospitalization when compared to women who do not use cocaine, though these data largely reflect adult populations^83^. Methamphetamines, though less common in younger teens, are strongly linked to ACS via catecholamine surge and vasoconstriction^79^. High-caffeine energy drinks, popular among adolescents, may exacerbate hypertension and trigger arrhythmias or QTc prolongation, especially when consumed in excess or with physical exertion^84^. These exposures are often underrecognized in clinical assessment but may contribute to early vascular injury and should be considered in cardiovascular risk screening.

E-cigarette use has grown rapidly among adolescent girls. While long-term outcome data are lacking, early studies show associations with increased blood pressure, vascular stiffness, arrhythmias, and endothelial dysfunction^85^. The AHA has emphasized concern about cardiopulmonary consequences and has called for more longitudinal data to assess cardiovascular outcomes in youth^86^.

### Reproductive-age women (20s–40s)

Cardiovascular disease in reproductive-age women is often underrecognized and misattributed, despite increasing evidence of unique pathophysiologic mechanisms, sex-specific risk factors, and distinct clinical presentations^2^.

#### Atherothrombosis

**Fig. 2:**
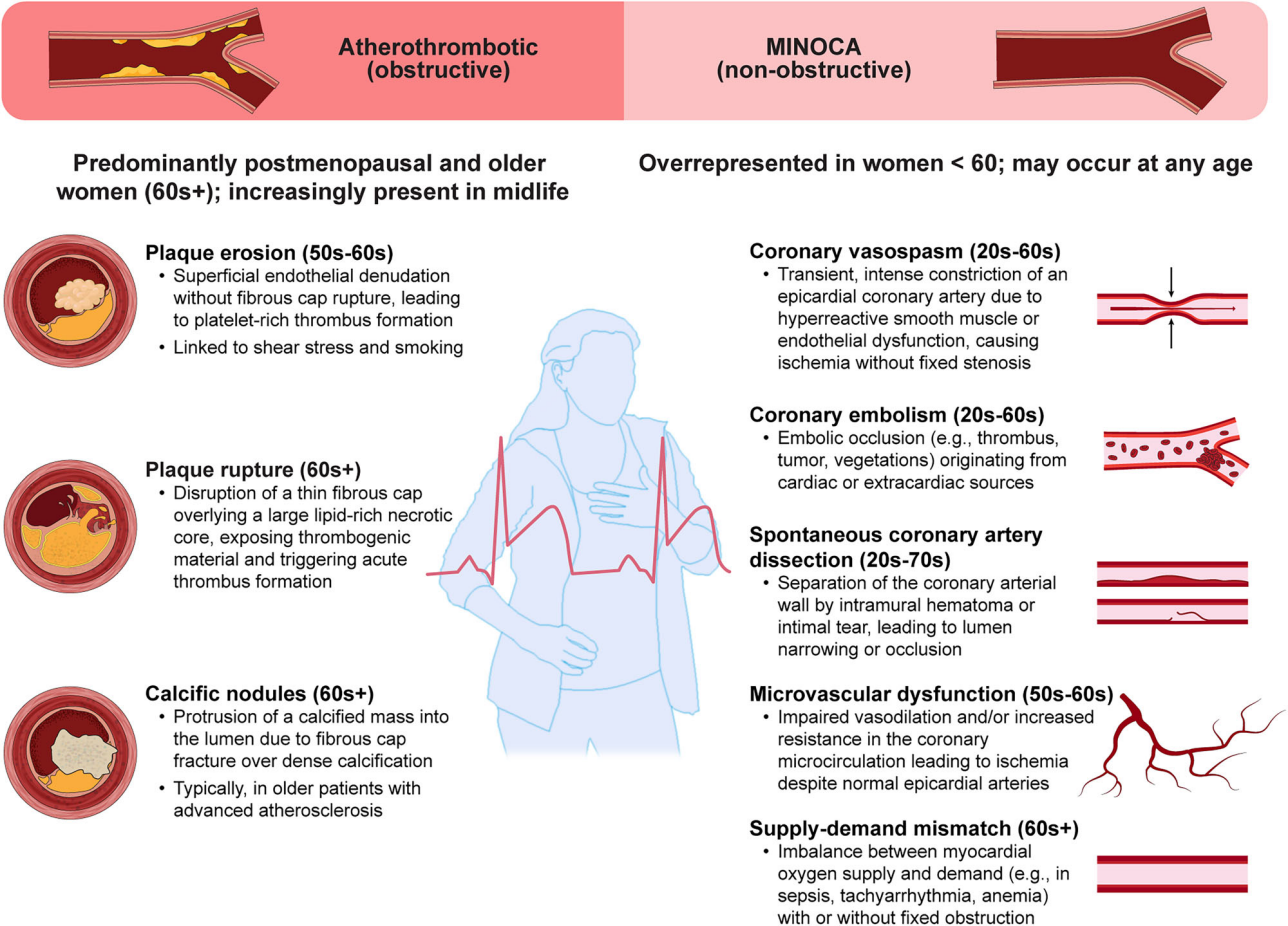
Pathophysiologic mechanisms of acute coronary syndromes in women. Women experience acute coronary syndrome (ACS) through both obstructive and non-obstructive mechanisms, with notable age-based variability. Atherothrombotic causes such as plaque rupture, erosion, and calcific nodules predominate in postmenopausal and older women, while non-obstructive causes, including spontaneous coronary artery dissection (SCAD), coronary vasospasm, microvascular dysfunction, and coronary embolism, are disproportionately represented in women under age 60. Mechanisms may coexist or evolve across the lifespan. MINOCA myocardial infarction with non-obstructive coronary arteries.

Atherothrombosis remains the leading cause of MI, even in reproductive-age women (Fig. 2). There is evidence of both a rising burden of atherothrombotic events and CVD mortality in middle-aged women and distinct mechanisms compared to men^3^. Coronary thrombosis occurs via plaque rupture, plaque erosion, or calcific nodules. Plaque rupture accounts for ~80% of fatal MIs in men but only ~60% in women^87^. Plaque erosion appears more common in women, particularly younger and premenopausal women, suggesting a possible protective role of estrogen against plaque rupture (which is uncommon in young women)^88^. Notably, a history of preeclampsia has been associated with increased CVD risk and a specific predisposition toward plaque erosion^89^.

Familial hypercholesterolemia (FH) is a major driver of early-onset CAD in women. Among those with an FH phenotype, women tend to experience earlier onset of ASCVD than men, with risk accelerated by 20–30 years^90^. Delayed diagnosis and treatment, along with pregnancy-related interruptions in statin therapy, contribute to worse outcomes^91^. Women with untreated FH face a 10–20-fold higher ASCVD risk and up to 30% chance of MI before age 60^92^.

#### Myocardial infarction with non-obstructive coronary arteries (MINOCA)

Myocardial infarction with non-obstructive coronary arteries (MINOCA) is a heterogeneous clinical syndrome occurring in approximately 5-15% of patients presenting with acute MI, more frequently in women^6^. By definition, MINOCA involves evidence of MI with no obstructive CAD (<50% stenosis) on angiography. Pathophysiologic mechanisms include plaque disruption with distal embolization, coronary vasospasm, SCAD, coronary microvascular dysfunction, and coronary thromboembolism. Sex-specific factors, including endothelial dysfunction, hormonal influences, and differential vascular responses, may contribute to the higher prevalence of MINOCA in women. Accurate diagnosis requires exclusion of alternate non-ischemic causes of troponin elevation (e.g., myocarditis, Takotsubo syndrome, arrhythmias) and often relies on adjunctive testing such as intracoronary imaging and especially early cardiac MRI^93,94^. In a community cohort retrospectively utilizing all clinical data among patients with troponin elevation, including coronary angiography, the rate of truly unclassifiable MINOCA in women aged 65 or younger was only 3%^7^.

#### Spontaneous coronary artery dissection

SCAD occurs disproportionately in women (85–90% of cases), and while it accounts for a minority of ACS overall, approximately 10% of MIs in women under age 65, up to a quarter under age 55, and 40% of pregnancy-associated MI cases are due to SCAD, highlighting its disproportionate impact in these populations^4,7,95^. SCAD results from an intramural hematoma or intimal tear that separates arterial wall layers, causing luminal compression or an obstructing flap. The identification and diagnosis of SCAD by coronary angiography can be overlooked when only subtle evidence is seen. Increasing awareness of this entity, particularly in young women, and intracoronary imaging such as intravascular ultrasound (IVUS) or optical coherence tomography (OCT) have increased recognition in ACS patients^4^.

SCAD^4^ is associated with several non-atherosclerotic arteriopathies, including fibromuscular dysplasia (present in 40–80% of cases), coronary artery tortuosity, and, rarely, connective tissue disorders like Marfan or Ehlers-Danlos syndromes (seen in up to 5% of cases)^96^. Pregnancy-associated SCAD is often more severe, with multivessel or left main involvement^96^. Hormonal influences have been hypothesized, but the occurrence of SCAD in nulliparous and postmenopausal women suggests multifactorial mechanisms. Triggers are noted in up to 25% of patients and include extreme physical exertion and Valsalva-like maneuvers (e.g., retching, coughing), and emotional stress^97^.

#### ACS due to coronary artery emboli

Embolic MI is also uncommon (2–5% of ACS cases), but important in younger women due to the frequent use of systemic hormonal contraceptives, which can predispose to thrombus formation^98^. Atrial fibrillation is the most common cause of coronary embolus overall, but other contributors include cardiomyopathies, prosthetic valve dysfunction, infective endocarditis, hypercoagulable states (de novo arterial thrombi and venous thrombi with paradoxical embolization), and cardiac tumors such as papillary fibroelastomas (PFE)^98^. These disorders should be considered in young women with MI in the absence of obstructive disease at coronary angiography^52^. Embolic MI is associated with higher morbidity and mortality, including increased risk of recurrent thromboembolism, MACE, and early mortality, especially in the context of atrial fibrillation^99,100^. Women with embolic MI may present at a younger age and with fewer traditional risk factors^52^. Prompt recognition and systematic evaluation for embolic sources are critical for appropriate management and secondary prevention. If paradoxical embolization is on the differential, evaluation to rule out an intracardiac shunt, including a patent foramen ovale, should be performed, with closure if clinically relevant.

Thrombophilia affects men and women equally, but hormonal states (pregnancy, oral contraceptives, hormone therapy) amplify thrombotic risk in women^101^. An inherited thrombophilia (e.g., factor V Leiden, prothrombin G20210A) confers a higher odds ratio for acute MI in young women compared to men^102^. Antiphospholipid syndrome, more common in women, confers particularly high thrombotic risk^103^.

PFEs are more frequently found in women, with large surgical and echocardiographic series consistently reporting a female predominance of over 60%^104^. While the primary clinical risk of PFEs is embolic stroke, embolic MI rarely occurs, typically in the context of left-sided, mobile, or aortic valve PFEs^104^.

#### Coronary vasospasm and microvascular dysfunction

Coronary vasospasm and microvascular dysfunction are closely linked and disproportionately affect women, especially those with MINOCA or INOCA^29^. It is estimated that 30–50% of patients with INOCA have microvascular dysfunction, especially women under 50 years of age^6^. In the WISE study, nearly two-thirds of women with MINOCA as the cause of ACS had microvascular dysfunction identified on stress cardiac magnetic resonance imaging, and up to one-third of women with non-obstructive CAD had vasospasm detected by acetylcholine provocation testing^105,106^. These entities contribute significantly to angina and ischemia in young women and are often underdiagnosed.

Importantly, microvascular dysfunction can be both a cause and a consequence of ischemia, which complicates risk stratification. It is also highly prevalent and associated with a worse prognosis among women with heart failure with preserved ejection fraction^107^. These mechanisms highlight the need for advanced diagnostic evaluation and sex-specific management strategies.

#### Peripartum and pregnancy-associated ACS

Cardiovascular disease is the leading cause of pregnancy-associated mortality in the United States, with ACS representing a growing proportion of these events. The risk of MI increases 3-fold during the peripartum period, with the highest incidence in the third trimester and early postpartum^108^. The incidence of pregnancy-associated MI is estimated at 2.8–8.1 per 100,000 deliveries, with maternal mortality rates ranging from 4.5 to 7.3%^109^.

According to a recent population-based study, most pregnancy-associated ACS are non-atherosclerotic in origin. SCAD is the most common mechanism, accounting for up to 44% of cases^110^. Other etiologies include atherosclerosis, coronary embolism, coronary thrombosis (often without underlying plaque), and coronary vasospasm. Sometimes, the coronary arteries appear normal on imaging, such that the etiology is uncertain. The hypercoagulable state of pregnancy, characterized by increased procoagulant factors, reduced fibrinolysis, and acquired protein C resistance, further contributes to thrombotic risk^108^. These changes are hormonally mediated, present early in gestation, and persist postpartum.

Hypertensive disorders of pregnancy, particularly preeclampsia and HELLP (hemolysis, elevated liver enzymes, low platelet count) syndrome, are common CVD risk enhancers^111,112^. Preeclampsia is associated with a 71% increase in cardiovascular mortality and a two- to four-fold increase in long-term CAD, heart failure, and stroke^112^. These outcomes are mediated in part by the subsequent development of chronic hypertension, diabetes, and dyslipidemia^111^. Low-dose aspirin is recommended in high-risk pregnancies to reduce preeclampsia risk^113^. Additional prevention strategies include lifestyle changes targeting regular exercise, smoking cessation, and balanced dietary intake^114^. Importantly, Black women in the U.S. are disproportionately affected by hypertensive pregnancy disorders and peripartum cardiovascular complications^22^. Biological factors, such as genetic variants, may also contribute to this increased risk, in addition to systemic inequities and healthcare disparities.

Women with prior ACS, whether due to atherosclerosis, SCAD, or other causes, are considered high risk for subsequent pregnancy-related cardiovascular events. Women with prior MI should be counseled that pregnancy may require medication adjustment and, in itself pose increased risk, especially in the early postpartum period^4,101,115^. Shared decision-making, ideally within a multidisciplinary cardio-obstetrics team, is critical to determine whether and when pregnancy is advisable. For instance, DAPT can be associated with increased maternal and fetal bleeding^101^. In most cases, it is recommended to complete the necessary DAPT course prior to conception. If pregnancy occurs while on DAPT, decisions must be individualized in consultation with cardiology, maternal-fetal medicine, and hematology specialists. Aspirin is generally considered safe in pregnancy and may be continued. The decision to continue or modify DAPT should be made on a case-by-case basis, balancing maternal cardiovascular risk and fetal safety, and planning for delivery and anesthesia^115^.

#### Contraception and hormone therapy

Combined hormonal contraceptives (CHCs) increase the risk of venous thromboembolism (VTE) by seven- to eight-fold, with risk varying by estrogen dose and progestin formulation^116^. Since non-oral systemic hormonal formulations bypass first-pass metabolism of the liver, avoiding the activation of clotting factors, triglycerides, and inflammatory markers, as seen with oral estrogen, these have been considered a safer option. However, some reports have shown non-oral formulations confer similar risk, perhaps by other mechanisms^117^. Progestin-only contraceptives (POCs), such as the levonorgestrel intrauterine device (IUD) or progestin-only pills, do not increase VTE risk, with the exception of injectable medroxyprogesterone acetate, which may have a modest effect^118^. IUDs, including copper and levonorgestrel-releasing types, do not increase cardiovascular risk and are considered safe for women with or at risk for cardiovascular disease, including those on anticoagulation^119^.

Women with antiphospholipid syndrome, SLE, or inherited thrombophilia should avoid CHCs. In these cases, POCs or non-hormonal methods are preferred, as supported by the American College of Radiology and ACC/AHA consensus^120^.

### Peri- and early postmenopausal women (50s–60s)

The perimenopausal and early postmenopausal years represent a pivotal stage in the emergence of ischemic heart disease in women. This period is marked not only by increasing cardiovascular risk but also by changing disease patterns and diagnostic challenges. For instance, the use of systemic menopausal hormone therapy (MHT) can increase the risk of VTE, stroke, and possibly coronary events. As such, MHT is not routinely recommended for the primary or secondary prevention of CVD. However, when initiated in healthy women younger than 60 years or within 10 years of menopause onset, MHT appears to be associated with low CV risk and may confer cardiometabolic benefits^121^. In contrast, MHT initiation later in life or in women with established atherosclerosis is associated with greater adverse cardiovascular outcomes. Risk stratification based on age, time since menopause, and comorbidities is essential. Nonsystemic, topical MHT, such as vaginal estrogen do not have systemic effects and is safe to use, especially for genitourinary symptoms^122^. For many women, this is also a life stage that may include changing demands at work, musculoskeletal issues, and associated decreased exercise, or new caregiving responsibilities for aging parents, all of which may adversely affect cardiovascular health and contribute to the upward inflection of cardiovascular risk factors, particularly decreased physical activity, weight gain, and increasing blood pressure.

#### Atherosclerotic ACS

Compared with men, women in this age group experience a delayed onset of obstructive ACS, on average, a decade later, but the delay is paralleled by a lag in recognition and diagnosis. Clinical presentation may be subtle, with symptoms such as exertional fatigue, nausea, sleep disturbance, or dyspnea being more prominent than chest pain. These patterns contribute to diagnostic uncertainty, delayed triage, and lower rates of guideline-based therapies at presentation^11,14^.

#### Menopause, the clinical and risk factor inflection point

Many women first develop overt coronary symptoms or events during the menopausal stage. The menopause transition is associated with adverse changes in lipids, body fat distribution, and vascular function, which accelerate cardiovascular risk and often coincide with the initial presentation of coronary symptoms in women aged 50–60 years^26^.

A substantial proportion of women in this age group have angina with normal or minimally diseased epicardial coronary arteries^123^. These women are at increased risk for adverse outcomes, including recurrent hospitalizations and higher mortality, underscoring that angina and/or ischemia without obstructive disease is not benign^123^. Clinicians should maintain a high index of suspicion for ischemic heart disease in symptomatic menopausal women, even in the absence of obstructive epicardial disease, and pursue appropriate diagnostic and management strategies^14^.

#### Non-obstructive ACS

Women in their 50s and early 60s are disproportionately affected by MINOCA, which accounts for approximately 6–15% of all MI presentations in women, with higher prevalence compared to men^6^. Common mechanisms include plaque erosion, SCAD, vasospasm, and thromboembolism, discussed above. Microvascular dysfunction, a leading cause of exertional chest pain and reduced quality of life in middle age in older women, may rarely cause ACS, but most often presents with exertional angina, abnormal stress testing, and normal or near-normal coronary imaging^45^. These etiologies may be missed or miscategorized with coronary angiography alone, necessitating advanced intracoronary imaging such as optical coherence tomography (OCT) or intravascular ultrasound (IVUS) and early cardiac magnetic resonance (CMR) to clarify the underlying cause^94,124^.

#### Older women (60s+)

In later life, the burden of atherosclerotic CVD becomes increasingly pronounced in women. However, the clinical expression of ACS in older women often diverges from textbook paradigms, with important implications for recognition, management, and outcomes.

#### Atherosclerotic ACS in the setting of multimorbidity

Atherosclerotic ACS is the predominant phenotype in women over age 60, often occurring in the setting of multiple comorbidities such as hypertension, diabetes, chronic kidney disease, and anemia. These coexisting conditions contribute to atypical presentations, higher procedural risk, and more complex medical management when compared to younger individuals^125^. Despite similar plaque burden compared to men, older women are less likely to undergo early invasive evaluation or receive guideline-directed medical therapy at discharge, even when presenting with high-risk features^125^. Provider bias, both conscious and unconscious, also plays a significant role, as clinicians are less likely to recommend invasive evaluation for women, even when clinical vignettes are identical to those of men^126^. Contemporary registry data also suggest that sex differences can extend into the post discharge period; in the PRAISE registry, women had different post discharge clinical profiles and outcomes compared with men, with differences attenuated after multivariable adjustment^127^.

When revascularization is pursued, women have higher rates of vascular complications and bleeding. This is attributed to smaller vessel caliber, older age, higher prevalence of anemia and renal dysfunction, and increased sensitivity to antithrombotic agents^128^. Women may derive a relatively greater absolute benefit from radial access in the setting of increased vascular access complications; however, the guideline recommendations to prioritize radial access are not sex specific^129,130^. Furthermore, women have smaller coronary arteries, which present additional technical challenges during PCI and may impact long term results^131^. Older women may also derive less symptomatic benefit from PCI in the presence of diffuse or microvascular disease, which is more prevalent in this patient population group^131^.

#### Under-recognition of symptoms

Women who are 75 or older more frequently present with multiple and less obvious ischemic symptoms, including weakness, confusion, nausea, or falls, rather than chest pain^128^. This symptom profile, coupled with age- and gender-based biases, contributes to diagnostic delay and lower likelihood of receiving reperfusion therapy in ST-elevation ACS^128^. Cognitive impairment, sensory limitations, and social isolation further complicate symptom recognition and timely presentation^125^.

#### Frailty, disability, and polypharmacy

Frailty, defined as reduced physiologic reserve and increased vulnerability to stressors, affects a substantial proportion of older women with ACS and is independently associated with higher mortality, bleeding, and poor functional recovery^125,132^. Disability, both pre-existing and acquired post-ACS, further complicates recovery, limits participation in cardiac rehabilitation, and increases the risk of long-term institutionalization^125^. Polypharmacy, common in this population, raises the risk for adverse drug interactions, medication non-adherence, and cognitive side effects^125^. Importantly, clinical trial data guiding ACS therapy in frail, disabled older adults, particularly women, are limited, necessitating individualized, goal-aligned care that balances longevity with functional outcomes and quality of life. Patient-centered approaches are essential in this population, ensuring that treatment decisions align with individual goals, preferences, and functional priorities, particularly in the context of multimorbidity and age-related complexity^133^.

### Consideration for antiplatelet therapy after ACS in women

Sex-specific considerations are critical in the use of DAPT for women with ACS, particularly those of reproductive age. Women have a higher prevalence of non-atherothrombotic ACS mechanisms and are at greater risk for bleeding complications, including menorrhagia and peripartum hemorrhage^134^. Sex-specific data on DAPT duration and selection are limited, especially in patients with MINOCA, SCAD, and cancer- or autoimmune-related ACS, where pathophysiology may not require prolonged platelet inhibition. A 2025 clinical consensus statement of the European Association of Percutaneous Cardiovascular Interventions and the ESC Working Group on Thrombosis identifies strategies to improve the safety and efficacy of antithrombotics in women with ACS. The authors highlight the underrepresentation of women and sex-based analyses in current research and emphasize the importance of including women in randomized controlled trials so that sex-based analyses and recommendations can be sufficiently performed^130^.

In women with SCAD, conservative management is preferred in clinically stable patients, given the high rate of spontaneous healing and the elevated procedural risks associated with PCI^4^. Once SCAD is confirmed, anticoagulation initiated for ACS is usually discontinued unless otherwise indicated. For those managed without revascularization, antiplatelet therapy practices vary and are an active area of investigation. While single antiplatelet therapy with aspirin is commonly used, the optimal regimen and duration remain unproven. Observational data suggest that DAPT, especially with more potent P2Y12 inhibitors, may be associated with a higher risk of early adverse events in conservatively managed SCAD, supporting individualized, often restrained use of DAPT in this context^135,136^. In contrast, when PCI is performed for SCAD, DAPT is indicated; however, duration should be tailored to bleeding risk and coexisting atherosclerosis^130^. In select patients, shorter DAPT (1–3 months) followed by P2Y_12_ monotherapy may be reasonable, though evidence is extrapolated from non-SCAD PCI trials. Similarly, the role of DAPT in MINOCA and INOCA remains poorly defined and should be tailored to the suspected mechanism^6,130^.

### Cardiac rehabilitation in women across the lifespan

Cardiac rehabilitation (CR) is a Class I recommendation endorsed by the AHA, ACC, and partnering societies for all eligible patients following ACS^126,137^. Despite these benefits, women remain underrepresented in every stage and every age of CR utilization, especially among very elderly and reproductive age women. Sociocultural and structural factors compound these gaps. Women are more likely to serve as primary caregivers and may prioritize the health of others over their own. Financial constraints, limited insurance coverage, and logistical challenges such as transportation or time off work reduce participation. Women of lower socioeconomic status and those without insurance are significantly less likely to attend CR; insured patients are three times more likely to be referred.

A recent multi-society endorsed AHA scientific statement emphasizes that CR improves women’s functional status, reduces hospital readmissions, lowers mortality, and enhances quality of life. The authors emphasize the need to improve referral and participation among women and to provide gender and culturally sensitive CR programming that is tailored to the individual woman’s unique physiological, psychological, and social needs, as well as ACS diagnosis. Improving the effectiveness and participation of CR will require employing non-traditional models that emphasize mental health, address sex differences in physiology, and gender differences in priorities, and for clinicians to emphasize its importance to their patients.

## Conclusion

ACS in women arise from a complex interplay of sex-specific, age-related, and systemic factors, demanding nuanced clinical attention across the lifespan. In pediatric and adolescent girls, early exposures such as congenital coronary and cardiac anomalies, autoimmune conditions, and substance use set the stage for later cardiovascular risk. During reproductive years, hormonal fluctuations, pregnancy, autoimmune and inflammatory disorders, and SCAD contribute to non-traditional ACS presentations. The perimenopausal period marks a critical transition, with rising prevalence of atherosclerotic ACS, often compounded by microvascular dysfunction and cancer therapy-related ischemia. In older women, multimorbidity, frailty, and under-recognition of symptoms dominate the clinical picture, leading to delayed diagnosis and suboptimal care. Tailored prevention, diagnostics, and therapies are urgently needed, and clinicians must adopt a sex- and age-informed lens with shared decision-making. There is also a critical need for sex-based prospective studies.

A lifespan-based approach to ACS in women is not only evidence-informed but essential to delivering equitable cardiovascular care. This review highlights the urgent need for an equitable approach to assessment for ACS in women, tailored prevention and treatment of CVD, and inclusion of women with robust sex-based exploration of diagnostic mechanisms and therapeutic measures for ACS in current research.
